# Transcranial direct current stimulation enhances the protective effect of isoflurane preconditioning on cerebral ischemia/reperfusion injury: A new mechanism associated with the nuclear protein Akirin2

**DOI:** 10.1111/cns.70033

**Published:** 2024-09-12

**Authors:** Xiangyi Kong, Wenyuan Lyu, Xiaojie Lin, Hao Feng, Lin Xu, Chengwei Li, Xinyi Sun, Chunlong Lin, Jianjun Li, Penghui Wei

**Affiliations:** ^1^ Department of Anesthesiology, Qilu Hospital (Qingdao), Cheeloo College of Medicine Shandong University Qingdao China; ^2^ Laboratory of Anesthesia and Brain Function, Qilu Hospital (Qingdao), Cheeloo College of Medicine Shandong University Qingdao China

**Keywords:** Akirin2, ischemic stroke, isoflurane preconditioning, oxidative phosphorylation, tDCS

## Abstract

**Aims:**

Ischemic stroke is a major cause of disability and mortality worldwide. Transcranial direct current stimulation (tDCS) and isoflurane (ISO) preconditioning exhibit neuroprotective properties. However, it remains unclear whether tDCS enhances the protective effect of ISO preconditioning on ischemic stroke, and the underlying mechanisms are yet to be clarified.

**Method:**

A model of middle cerebral artery occlusion (MCAO), a rat ischemia–reperfusion (I/R) injury model, and an in vitro oxygen–glucose deprivation/re‐oxygenation (O/R) model of ischemic injury were developed. ISO preconditioning and tDCS were administered daily for 7 days before MCAO modeling. Triphenyltetrazolium chloride staining, modified neurological severity score, and hanging‐wire test were conducted to assess infarct volume and neurological outcomes. Untargeted metabolomic experiments, adeno‐associated virus, lentiviral vectors, and small interfering RNA techniques were used to explore the underlying mechanisms.

**Results:**

tDCS/DCS enhanced the protective effects of ISO pretreatment on I/R injury‐induced brain damage. This was evidenced by reduced infarct volume and improved neurological outcomes in rats with MCAO, as well as decreased cortical neuronal death after O/R injury. Untargeted metabolomic experiments identified oxidative phosphorylation (OXPHOS) as a critical pathological process for ISO‐mediated neuroprotection from I/R injury. The combination of tDCS/DCS with ISO preconditioning significantly inhibited I/R injury‐induced OXPHOS. Mechanistically, Akirin2, a small nuclear protein that regulates cell proliferation and differentiation, was found to decrease in the cortex of rats with MCAO and in cortical primary neurons subjected to O/R injury. Akirin2 functions upstream of phosphatase and tensin homolog deleted on chromosome 10 (PTEN). tDCS/DCS was able to further upregulate Akirin2 levels and activate the Akirin2/PTEN signaling pathway in vivo and in vitro, compared with ISO pretreatment alone, thereby contributing to the improvement of cerebral I/R injury.

**Conclusion:**

tDCS treatment enhances the neuroprotective effects of ISO preconditioning on ischemic stroke by inhibiting oxidative stress and activating Akirin2‐PTEN signaling pathway, highlighting potential of combination therapy in ischemic stroke.

## Graphical abstract

Transcranial direct current stimulation treatment alleviates oxidative stress and activates Akirin2‐PTEN signaling pathway, and enhances the neuroprotective effect of isoflurane pretreatment on ischemic stroke, highlighting the potential of combination therapy for ischemic stroke.

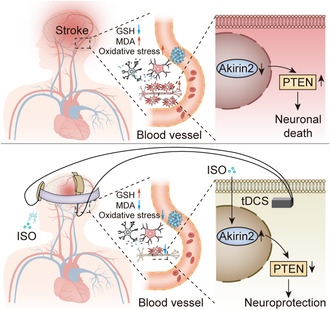

## 1 INTRODUCTION

Ischemic stroke is characterized by a blood clot occluding cerebral vessels that causes acute insufficient blood supply to the brain and resulting oxygen and glucose deprivation of neurons, which may lead to severe or fatal outcomes in patients.^1^ The prevalence and incidence of stroke are increasing annually, and the overwhelming majority of stroke cases, approximately 87%, is of ischemic nature.^2^ The etiology includes embolism, thrombosis, and decreased perfusion.^3^ With an aging surgical population, the incidence of ischemic stroke is increasing. Currently, there are still few effective interventions for ischemic stroke.^4^

Isoflurane (ISO), a widely used volatile inhalation anesthetic in the perioperative period for several decades, increases cerebral blood perfusion by inducing cerebral vasodilation.^5^ Clinical evidence has demonstrated that patients undergoing general anesthesia with inhaled anesthetics such as ISO and sevoflurane experienced lower rates of perioperative ischemic stroke in a dose‐dependent manner.^6^ Additionally, higher concentrations of ISO were found to have a significant protective effect on ischemic stroke within 30 days after surgery.^6^ Preclinical studies have also indicated that ISO may reduce infarct volume and enhance neurological recovery in cerebral ischemia–reperfusion (I/R) injury models.^7^, ^8^ Nevertheless, the short treatment duration of ISO limits its therapeutic effect; therefore, combination therapy is suggested.^8^ Transcranial direct current stimulation (tDCS) modulates neuronal excitability and promotes stroke recovery through non‐invasive brain stimulation.^9^, ^10^ Axonal regeneration and neuronal growth can also be promoted by direct current (DC) electric fields in tDCS‐induced neuroprotection. A previous study showed that DC can direct the migration of neural stem/progenitor cells in vitro,^9^ thus showing promise in treating several neurological diseases. Due to the complementary nature of their neuroprotective mechanisms, tDCS is expected to enhance the neuroprotective efficacy and consistency of ISO.

Akirin is a newly identified nuclear protein, and it includes two highly evolved and conserved nuclear factors Akirin1 and 2.^11^ Recent studies are uncovering their roles in multiple developmental processes as critical transcriptional regulators.^11^, ^12^ Akirin2 is required in embryonic development and innate immunity.^13^ Loss of Akrin2, and not Akrin1, is embryonically lethal in mice.^14^ Akirin2 promotes angiogenesis in cholangiocarcinoma through IL‐6/STAT3/VEGFA signaling pathway.^15^ Akirin2 is overexpressed in primary glioblastomas in humans.^13^ Akirin2 plays an essential role in the formation of the cerebral cortex and promotes neuronal differentiation at the neurogenin‐related‐1 level in *Xenopus*.^16^, ^17^ The emerging evidence suggests a correlation between Akirin2 dysfunction and neurological disorders.^18^, ^19^ The role of Akirin2 in ischemic stroke is yet to be explored.

In this study, we investigated the therapeutic potential of ISO preconditioning in ischemic stroke using in vivo and in vitro cerebral I/R injury models. Next, we explored the synergistic effect of ISO and tDCS combination therapy and further determined whether the underlying mechanism is associated with Akirin2‐related pathways. A deeper understanding of the mechanisms underlying ISO and tDCS combination therapy may present new therapeutic opportunities in recovery of ischemic stroke.

## 2 MATERIALS AND METHODS

### 2.1 Animals

All animal experiments were approved by a committee at Qilu Hospital (Qingdao) (KYDWLL‐202105), and we followed all guidelines for the use of laboratory animals by the Institutional Animal Care and Use Committee at Shandong University. We selected 6‐week‐old male Sprague–Dawley (SD) rats weighing between 250 and 300 g, housed in an environment maintained at 23–25°C with a 12‐h light–dark cycle. The experimental study was carried out after acclimating the rats for a week. In this experiment, data collection and samples were divided into blind groups only, and no personnel knew the composition of the experimental groups.^20^ The figure legends specified the number of rats that contributed data for analysis in each experiment.

### 2.2 Transient focal cerebral ischemia modeling

Our previous study established a transient focal cerebral ischemia rat model using middle cerebral artery occlusion (MCAO).^21^ The MACO procedure was conducted under 3.5%–4% ISO anesthesia, with spontaneous ventilation maintained using an oxygen mask during surgery.^22^ The temperature of the rats was maintained by heating blankets (37.0 ± 0.5°C) during the surgery. Monofilament nylon sutures (Jialing, China) were used to penetrate the right internal carotid artery from the right external carotid artery (ECA) and block the right middle cerebral artery (MCA). After 1 h of occlusion, the thread plug was removed and the ECA was ligated. In the sham group, thread pins were not inserted into the MCA. The rats were euthanized by decapitation under terminal anesthesia, and brain tissue samples were harvested 6 or 24 h after MCAO modeling.^23^

### 2.3 Triphenyltetrazolium chloride staining and measurements of the infarct volume

Triphenyltetrazolium chloride (TTC) staining (Solarbio, China; G3005) was performed to measure the infarct volume, as mentioned in our previous study.^24^ The rats were euthanized 24 h after reperfusion of MCAO, and the brain tissue was cut into 2 mm‐thick slices (unit: mm^3^) after harvesting the brain. The brain slices were stained with 2% TTC for 30 min and fixed in 4% paraformaldehyde (PFA) (Solarbio, China; P1110) for 1 day. Images were obtained using a scanner (LiDE 120; Jianeng, China). The infarct size was determined using Image J software. To calculate the infarct volume, the ischemic zone volume ratio was computed as follows: ischemic zone volume ratio = (sum of white ischemic zone area in each slice)/(sum of brain slice area in each slice) × 100%.^25^, ^26^

### 2.4 Behavioral test

The modified neurologic severity scores (mNSS) is a behavioral evaluation method used to assess motor function, sensory function measurement reflexes. The total score ranges from 0 to 14 (with a normal score of 0 and a maximum deficit score of 14).^27^ Hanging wire test was used to test the grip strength of rats. The rats were assessed using the grip strength test, wherein they were permitted to grasp the cage cover and invert it, and the duration for which their hind limbs remained suspended was recorded. Each rat was given a maximum of 120 s, after which it was removed. Before testing, all rats were acclimated to the behavioral chamber. The grip strength tests were conducted at 1, 7, and 14 days after MCAO induction in the rats.^28^

### 2.5 Primary cortical neuron culture

Cortical neurons were prepared from embryonic SD rats on day 17 of gestation following established protocols.^29^ The fetal brain tissue was harvested, the meninges were peeled off, and the remaining brain tissue was digested at 37°C for 20 min with 0.05% trypsin (Proteintech, China; PR40020). After filtration, the suspended neurons were cultured in petri dishes coated with poly d‐lysine, and the following ingredients were added to the neuronal medium: 95.5% Neurobasal medium (Gibco, USA; 2103049), 2% B‐27 additive (Gibco, USA; 17504044), 1% GlutaMAX (Gibco, USA; 35050061), 0.5% fetal bovine serum (Gibco; A5669701), and 1% Penicillin–Streptomycin Solution (Proteintech; PR40022). The neurons were cultured for 14 days before the experiment.

### 2.6 ISO preconditioning and induction

To precondition the rats, they were exposed to 1.4 vol% ISO along with 98% O2 (at a flow rate of 1.5 L/min) for 1 h per day for a week prior to the MCAO procedure.^30^ The rats received ISO pretreatment at the same time each day. The control group of animals were exposed to oxygen only for the same duration.^31^ For in vitro ISO preconditioning, approximately 1 × 10^6^ neurons were cultured in 1.5 mL of neural basal medium in six‐well plates. ISO (1.4%), oxygen (21%), and carbon dioxide (5%) were administered using an anesthesia machine into an incubator, with continuous monitoring of the concentrations using a Datex infrared gas analyzer. This procedure was based on a previous study to mimic in vitro ISO preconditioning.^32^

### 2.7 Oxygen–glucose deprivation/re‐oxygenation injury

We established an oxygen–glucose deprivation/re‐oxygenation (O/R) injury model according to our previously published protocol.^33^ The OGD model first involves adding deoxygenated extracellular solution (ECS) to the cells. This solution contains 116 mM NaCl, 5.4 mM KCl, 0.8 mM MgSO_4_, 1.0 mM NaH_2_PO_4_, 1.8 mM CaCl_2_, and 26 mM NaHCO_3_. The cells are then placed in a hypoxic incubator (37°C, 95% N_2_, and 5% CO_2_) for 1 h. Subsequently, the cells are switched back to the normal medium and transferred to a standard incubator. For the control group, ECS containing 116 mM NaCl, 5.4 mM KCl, 0.8 mM MgSO_4_, 1.0 mM NaH_2_PO_4_, 1.8 mM CaCl_2_, 26 mM NaHCO_3_, and 33 mM glucose is added to the cells. The control group is placed in a normal temperature incubator (37°C, 95% air) and cultured in 5% CO_2_ for 1 h. After this period, the cells are returned to the original maintenance medium. The experiment was carried out 6 h later.

### 2.8 Ultra‐high performance liquid chromatography‐mass spectrometry/mass spectrometry analysis

In this study, cerebral cortex tissue was harvested and weighed from the sham, MCAO, and ISO groups 6 h after MCAO surgery. Samples were placed in a 4°C automatic injector during the entire analysis process. The samples were analyzed by SHIMADZU‐LC30 ultra‐high performance liquid chromatography (UPLC) using the ACQUITY UPLC® HSS T3 (2.1 × 100 mm, 1.8 μm) (Waters, Milford, MA, USA) column. The sample size was 4 μL, the column temperature was 40°C, and the flow rate was 0.3 mL/min. Chromatographic mobile phase A comprised 0.1% formic acid solution, whereas phase B comprised acetonitrile. The chromatographic gradient elution procedure was as follows: 0–2 min, 0 B; 2–6 min, B changed linearly from 0% to 48%; 6–10 min, B changed linearly from 48% to 100%; 10–12 min, B was maintained at 100%; 12–12.1 min, B changed linearly from 100% to 0%; and 12.1–15 min, B remained at 0%.

Positive (+) and negative (−) modes of each sample were detected by electrospray ionization. The samples were separated by UPLC and analyzed by mass spectrometry using QE Plus mass spectrometer (Thermo Scientific). Ionization was performed using HESI source. The ionization conditions were as follows: spray voltage, 3.8 kv (+) and 3.2 kv (−); capillary temperature, 320 (±); sheath gas, 30 (±); Aux gas, 5 (±); probe heater temperature, 350 (±); S‐Lens RF level, 50; mass spectrum acquisition time, 15 min; parent ion scanning range, 75–1050 m/z; primary mass spectrometry resolution, 70,000@m/z 200; AGC target, 3e6; and primary maximum IT, 100 ms. Secondary mass spectrometry was performed according to the following conditions: Secondary mass spectrometry (MS2 scan) of the 10 highest intensity parent ions is triggered after each full scan, secondary mass spectrometry resolution: 17,500@m/z 200, AGC target: 1e5, Level 2 maximum IT: 50 ms, MS2 activation type: HCD, isolation window: 2 m/z, normalized collision energy (stepped): 20, 30, and 40.

### 2.9 Oxidative stress measurement

Enzyme activity and content were determined using glutathione (GSH) (Beyotime, China; S0053) and malondialdehyde (MDA) (Beyotime; S0131M) kits according to the manufacturer's instructions. After performing MCAO, equal amount of steel balls was added to the rat brain tissue and ground 10 times at 70 Hz, for 10 s each time. For primary neurons, 0.1 mL of lysis buffer per million cells were used. After centrifugation, the supernatant was mixed with the working liquid, and the absorbance was detected at 405 nm and 532 nm by an enzymoleter. GSH and MDA concentrations were calculated and expressed as nmol/mg protein.

### 2.10 Measurement of intracellular reactive oxygen species

Dichlorodihydrofluorescein diacetate (DCFH‐DA) (Beyotime; S0033M) was diluted in serum‐free medium at 1:1000 to a final concentration of 10 μM. The neuron culture medium was replaced with an appropriate volume of diluted DCFH‐DA. Next, it was incubated in a cell incubator at 37°C for 30 min. The neurons were imaged by confocal microscopy after being washed three times in serum‐free cell culture solution (Leica Camera, STELLARIS 5).

### 2.11 Mitochondrial function assays

We used JC‐1 (Beyotime; C2006) mitochondrial membrane potential (MMP) test kit and Mito‐Tracker Red CMXRos (Beyotime; C1049B) to detect MMP in neurons, according to the manufacturer's instructions. To explain briefly, the cells were washed with phosphate‐buffered saline (PBS) three times. The serum‐free culture medium was configured with JC‐1 or mito‐tracker working solution; the neurons were added to the incubator at 37°C for 30 min and washed again with PBS three times. Images were observed and preserved under a laser confocal microscope.

### 2.12 Quantitative real‐time PCR analysis

Total RNA was extracted from neurons and the rat cortex using trizol and ReverTra Ace®qPCR RT Master Mix with gDNA Remover (TOYOBO; fsq301) for reverse transcription. Real‐time PCR was performed using an ABI Q3 machine (GenStar). Using GAPDH as internal reference, the quantitative real‐time PCR (qRT‐PCR) primers of the gene were detected by 2 × RealStar Green Power Mixture. The primer sequences used in this study are as follows (from 5′ to 3′): Akirin2, F: TCTCCTCCCGTCTCACCACAG, R: AATCGGAACTACAACCTGGGTCTG; GAPDH, F: GCGTCGGTGTGAACGG ATTTGG, R: GCCGTGGGTAGAGTCATACTGGAAC; phosphatase and tensin homolog deleted on chromosome 10 (PTEN), F: TCATACCAGGACCAGAGGAAACC, R: TTGTCATTATCCGCACGCTCTATAC.

### 2.13 Western blotting

We established a protocol for western blotting based on a previous study.^34^ An equal amount of protein (40 μg) was separated by sodium dodecyl sulfate‐polyacrylamide gel electrophoresis and were transferred onto polyvinylidene fluoride membranes. Subsequently, the membranes were blocked with a rapid blocking solution (Proteintech; PR20034) at room temperature for 1 h. They were incubated overnight at 4°C with a primary antibody containing anti‐Akirin2 (rabbit, 1:500, PA5‐20608; Thermo Fisher, USA), anti‐PTEN (rabbit, 1:1000, 9188; Cell Signaling Technology, USA), and anti‐beta‐Actin (rabbit, 1:1000, 81115‐1‐RR; Proteintech). On the second day, horseradish peroxidase‐coupled secondary antibody was incubated for 1 h, and protein bands for chemiluminescence detection were performed.

### 2.14 Enzyme‐linked immunosorbent assay

Rat interleukin (IL)‐1β, IL‐6 and tumor necrosis factor‐α were analyzed as previously described according to the manufacturer's instructions (E‐EL‐R0012c, E‐EL‐R0015c, E‐EL‐R2856c; Elabscience, China).^34^

### 2.15 Immunofluorescent staining

The procedure for immunofluorescence staining was based on a previous study.^34^ Brain sections (40 μm) were incubated with 10% sheep serum for 1 h and combined with anti‐Akirin2 (rabbit, 1:500, PA5‐20608; Invitrogen), anti‐PTEN (rabbit, 1:500, ab137337; Abcam), anti‐NeuN (mouse, 1:500, ab104224; Abcam) or anti‐microtubule‐associated protein 2 (mouse, 1:500, ab254143; Abcam) and incubated overnight at 4°C. On the second day, a goat anti‐rabbit or got anti‐mouse IgG probe coupled with AF488 or AF647 was used for 1 h. Sections were incubated with 4′,6‐diamidino‐2‐phenylindole for nuclear staining after being washed in PBS, prior to laser confocal imaging. The neurons were cultured in a 24‐well culture plate containing round coverslip. Next, they were fixed with 4% PFA for 20 min, replaced with PBS containing 5% fetal bovine serum and 0.3% Triton X‐100 for 2 h, and incubated at 4°C with primary antibody (anti‐Akirin2, anti‐PTEN, and anti‐MAP2) overnight. After incubation with secondary antibodies (AF488‐ or AF647) for 1 h, confocal microscopic imaging was conducted using a Leica Camera, STELLARIS 5. The same parameter settings were applied to all images.

### 2.16 Cell culture and siRNA‐mediated interference

To silence gene expression, 20 nM small interfering RNA (siRNA) was transfected into the designated cells using the standard procedure of Lipofectamine RNAiMAX transfection reagent (Thermo Fisher Scientific), according to the manufacturer's instructions. Next, the cells were stimulated and harvested for further analysis 48 h after transfection. The siRNA target sequence of PTEN (RIBOBIO, China) used was GGGTAAATACGTTCTTCAT.

### 2.17 Lentiviral construction and orthotopic injection

Akirin2 knockdown lentivirus was purchased from Shengyuankemeng Co., Ltd. (Shanghai, China). The siRNA sequence used to knockdown Akirin2 was GGATTTCGACCCACTGCTTA, with a carrier number of pLVx‐shRNA2‐Zsgreen1‐T2A‐puro and a component sequence of U6‐MCS‐Zsgreeen‐T2A‐puro. In the in vivo experiments, stereotactic imaging was used to target the SD rat cortex for intracerebral virus injection at a rate of 0.2 μL/min. The injection site was positioned 0.3 mm anterior to the bregma and 3 mm lateral to the midline, at a depth of 2 mm from the skull surface. MCAO surgery was performed 21 days post‐injection. In the in vitro experiments, neurons were infected with lentivirus and incubated for 48 h before being used for subsequent experiments.

### 2.18 DCS and tDCS implementation

In the OGD model, DCS was applied to neurons cultured in a culture chamber using the method we described earlier. For DCS stimulation, silver/silver chloride electrodes in a beaker are connected to the medium pool on either side of the chamber using an agar salt bridge. In the control culture conditions, all parameters remained the same, except that DCS was not included. HEPES acid (20 mm) was supplemented into the medium, and the pH was adjusted to 7.4. At 3 h after OGD, the cells were exposed to DCS stimulation at a current intensity of 250 mV/mm for 20 min.

A constant current stimulator (Schneider Electronics, Gleichen, Germany) was used for tDCS without anesthesia.^35^ The currents were applied transcranial to the temporal areas, and electrodes were directly attached to the cranium to ensure a defined contact area. Seven days before the onset of ischemia, an epicranial electrode was attached to the skull via a tubular plastic jacket. Using nontoxic glass ionomer cement, we fixed a dorsolateral prefrontal cortex graft to each side of the cranium. Plastic jackets were filled with saline before stimulation. The electrode contact area toward the skull was 3.5 mm. A cathodal electrode was connected to the ischemic cortex, and an anodal electrode to the anodal terminal. In the rats, tDCS was delivered at 2.86 mA per cm^2^ and 100 μA per second. A cathodal electrode was connected to the ischemic cortex, and anodal electrodes were attached to the terminal. Rats underwent I/R were subjected to a total of eight stimulations per day for a week following daily ISO preconditioning: 10 min of stimulation, followed by a 3‐min rest period, and then an additional 10 min of stimulation.^36^

### 2.19 Lactate dehydrogenase release and CCK‐8 assay

To detect neuronal survival, we used CCK‐8 (Solarbio; CA1210) and lactate dehydrogenase (LDH) (Beyotime; C0017) kits. For the CCK‐8 assay, 10 μL of CCK‐8 reagent was added to the 96‐well plate pretreated with drugs. After incubation for 2 h, CCK‐8 absorbance was detected by molecular devices (Molecular Devices, USA) at 450 nm. LDH release test was measured at a 490 nm absorbance according to the kit instruction.

### 2.20 Statistical analysis

All data were subjected to statistical analysis using GraphPad Prism 8.0. Each animal and cell experiment was replicated more than three times to ensure reliability. The Shapiro–Wilk test was used to assess the normality of the data distribution. Results are presented as mean ± standard deviation after testing for normality. In cases where the data did not adhere to a normal distribution, nonparametric tests including the Mann–Whitney *U* and Kruskal–Wallis tests were conducted based on the respective groupings. Conversely, for data that met the criteria for a normal distribution, statistical significance for comparisons between two independent groups was determined using Student's *t*‐test. Differences among multiple groups were analyzed using either one‐way analysis of variance (ANOVA) with Tukey's post hoc analysis or two‐way ANOVA with Bonferroni post hoc testing, depending on the experimental design and variables involved.^37^ A significance threshold of *p* < 0.05 was applied for determining statistical significance in the analyses conducted.

## 3 RESULTS

### 3.1 tDCS enhanced the neuroprotective effects of ISO preconditioning on brain injury and improved neurological outcomes after cerebral I/R injury

**FIGURE 1:**
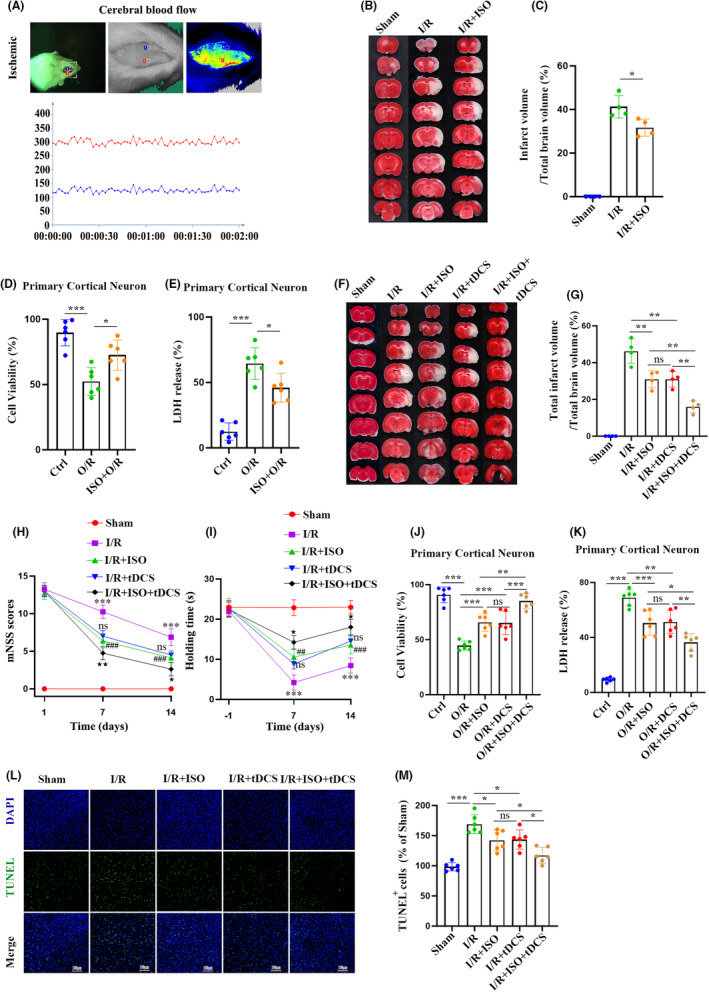
Transcranial direct current stimulation enhances the neuroprotective effect of isoflurane (ISO) after ischemia–reperfusion injury. (A) Representative two‐dimensional laser speckle images of rats with middle cerebral artery occlusion. (B, C) Triphenyltetrazolium chloride (TTC) staining images (B) and infarct volume statistics (C) of reduced infarct volume after ISO pretreatment (*n* = 4). Cell viability assay (D) and lactose dehydrogenase (LDH) release assay (E) were used to evaluate primary neuronal death after ISO pretreatment in oxygen–glucose deprivation/re‐oxygenation injury (O/R) (*n* = 6). TTC staining images (F) and statistics (G) of cerebral infarction volume in rats 24 h after ischemia–reperfusion (I/R) pretreatment with transcranial direct current stimulation (tDCS) and ISO (*n* = 4). The neurological deficit score (H) and grip strength (I) were used to evaluate neurological outcomes 7 and 14 days after I/R injury (*n* = 8), ***p < 0.001 vs. sham, ^##^ *p* < 0.01, and ^###^ *p* < 0.001 vs. I/R, I/R + tDCS vs. I/R + ISO no statistical significance, ^★^ *p* < 0.05, ^★★^ *p* < 0.01 vs. I/R + ISO, two‐way analysis of variance (ANOVA). Cell viability assay (J) and LDH release assay (K) were used to evaluate primary neuronal death after ISO and direct current stimulation pretreatment in oxygen–glucose deprivation (OGD) injury (*n* = 6). Confocal images of TUNEL (L) and statistics (M) in ipsilateral cerebral ischemic areas of rats treated with or without ISO (*n* = 6). The data are expressed as mean ± SD, *p < 0.05, **p < 0.01, and ***p < 0.001 by one‐way ANOVA or two‐way ANOVA. ns, non‐significant.

To determine whether ISO preconditioning can effectively attenuate cerebral I/R injury, rats were exposed to repeated low concentrations of ISO for 1 h daily for a week before transient focal cerebral ischemia modeling by MCAO. Infarct cerebral blood flow was detected to ensure the success of modeling in each rat using the laser speckle method (Figure 1A). The infarct volume of rats was assessed by TTC staining. Compared with the sham group, the cerebral infarction volume in the I/R group was significantly enlarged. ISO preconditioning reduced the average infarct volume (Figure 1B,C). A lower cell viability and higher LDH release were observed in the oxygen–glucose deprivation/re‐oxygenation (O/R group, compared with that in the control group. However, ISO treatment significantly reversed these changes (Figure 1D,E).

We next investigated the synergistic effect of tDCS and ISO pretreatment on brain I/R injury. tDCS alone had a similar therapeutic effect to ISO compared with ISO alone in reducing infarct size. However, as expected, the combination of tDCS and ISO significantly reduced infarct size in rats compared with ISO or tDCS alone (Figure 1F,G). mNSS and hanging wire test were used to evaluate neurological outcomes. Neurological deficit scores increased, and the grasping power assessed by hanging wire test decreased 7 and 14 days after MCAO. These ischemic effects were similarly attenuated by ISO or tDCS preconditioning (Figure 1H,I). Interestingly, tDCS significantly enhanced the ISO preconditioning‐mediated recovery of neurological deficit and neurological outcomes and decrease in neuronal apoptosis in the cerebral cortex (Figure 1H,I). Similarly, primary cortical neurons pretreated with DCS and ISO exhibited an increase in cell viability and a decrease in LDH release, compared with that of primary cortical neurons in the ISO group (Figure 1J,K). Moreover, TUNEL staining revealed more neuronal apoptosis in the cerebral cortex of rats with MCAO than that of sham rats (Figure 1L,M). This suggests that tDCS enhances the therapeutic effects of ISO preconditioning on I/R injury to attenuate severe brain injury and promote neurological recovery.

### 3.2 tDCS increased the inhibitory effect of ISO preconditioning on I/R‐induced oxidative phosphorylation

**FIGURE 2:**
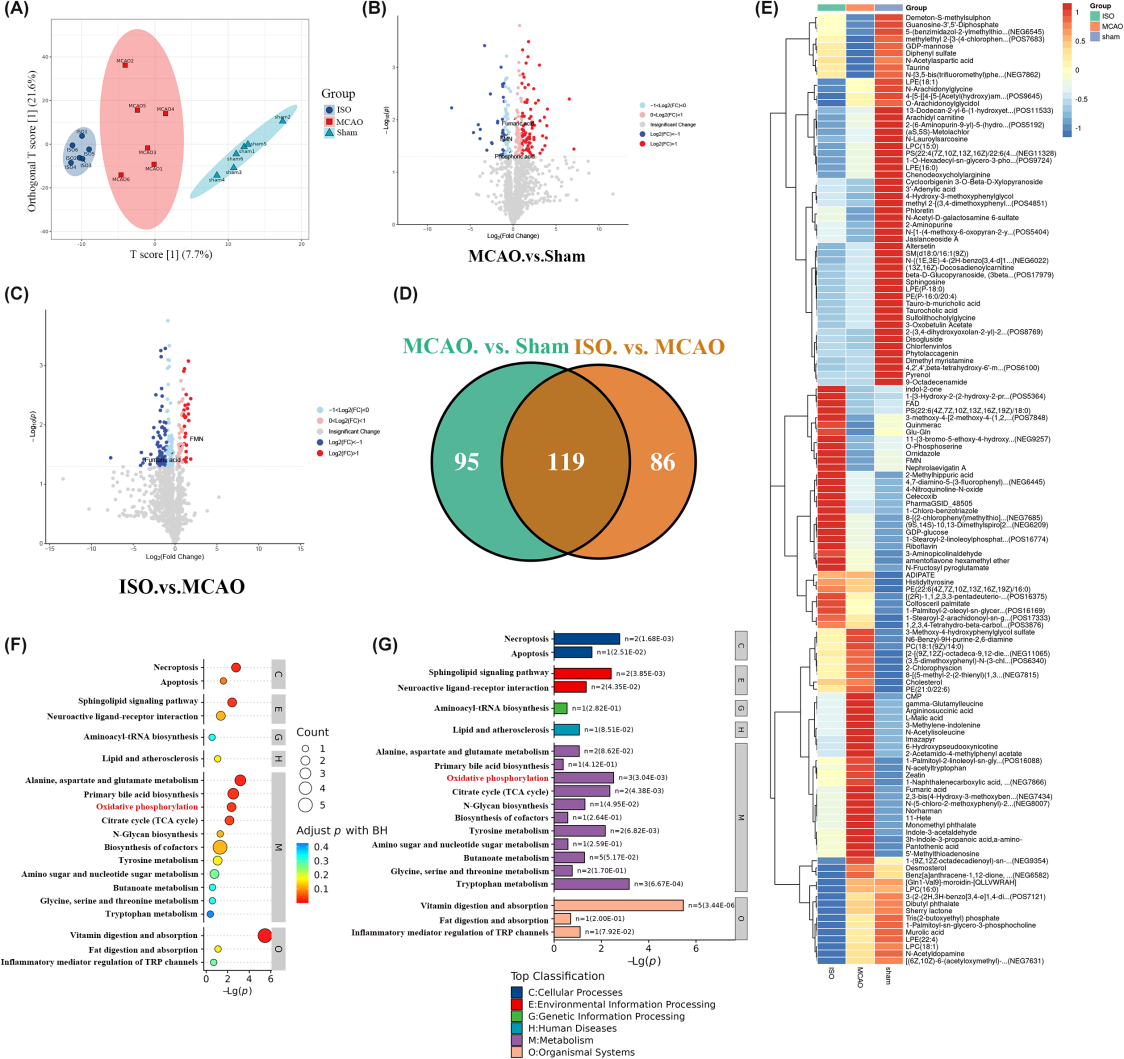
Isoflurane mediates cerebral ischemia–reperfusion injury in rats by modulating oxidative phosphorylation (OXPHOS). (A) The partial least squares discriminant analysis score map showed good separation between the sham, middle cerebral artery occlusion (MCAO), and isoflurane groups, suggesting that ischemia–reperfusion injury and isoflurane treatment resulted in cortical brain compensatory changes (*n* = 6). (B–E) We found 214 shared metabolites between the MCAO and sham groups and 205 shared metabolites between the isoflurane and MCAO groups; 119 shared metabolites were identified among the three groups and suggests that 119 differential metabolites mediate the neuroprotective effects of 1c treatment after ischemia–reperfusion injury. (F, G) The functions of these differential metabolites were acquired and enriched by Kyoto Encyclopedia of Genes and Genomes analysis and the results revealed that OXPHOS was significantly enriched.

**FIGURE 3:**
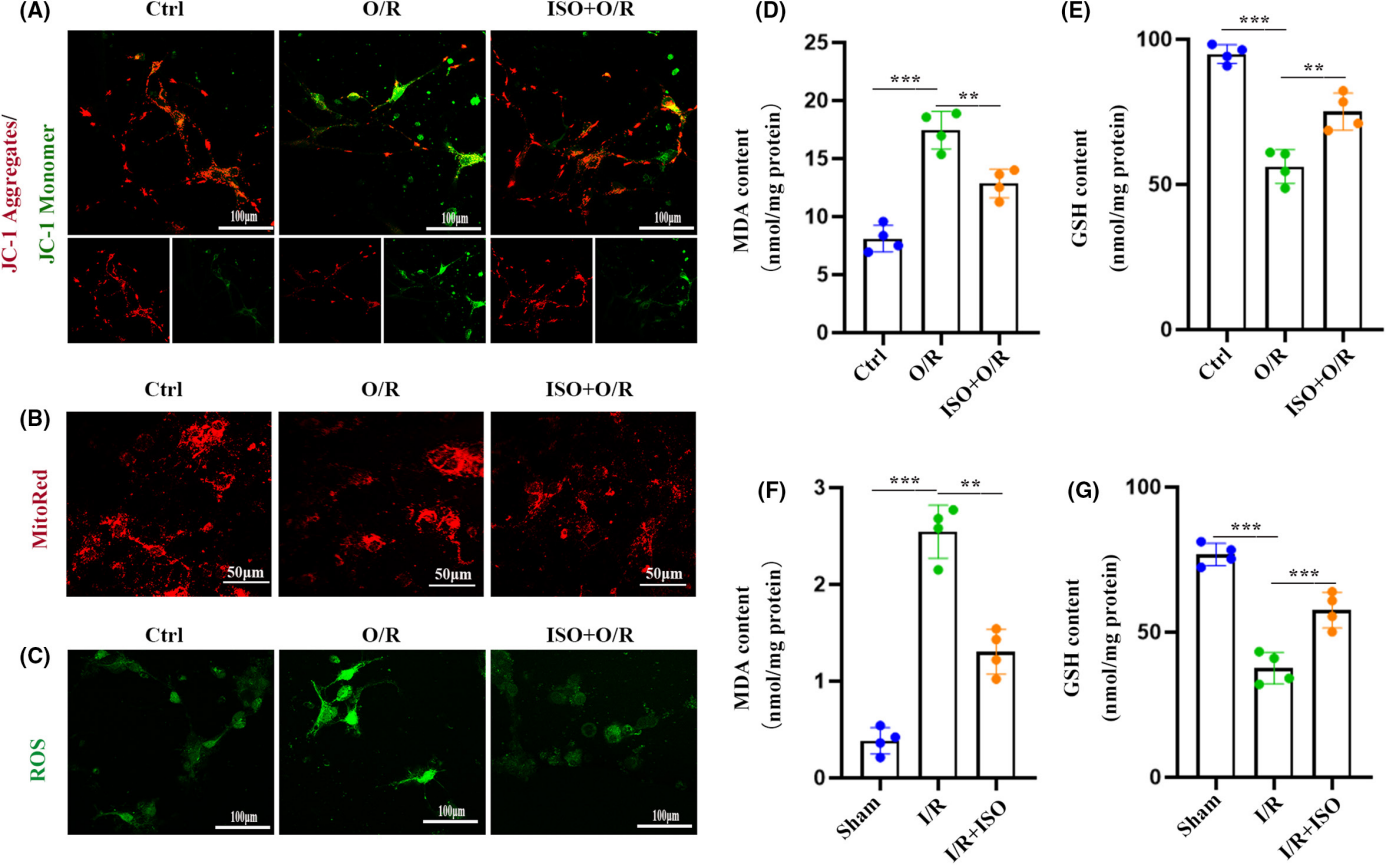
Isoflurane (ISO) alleviates cerebral ischemia–reperfusion injury in rats by inhibiting oxidative stress and neuroinflammation. (A) Using confocal neurons in JC‐1 aggregates (red) and monomers (green) (*n* = 4). (B) Mito‐Tracker red coloring shows a representative image of neurons with ISO pretreatment (*n* = 4). (C) The reactive oxygen species (ROS) levels of neurons labeled with DCFH‐DA fluorescent probes were analyzed by fluorescence microscopy (*n* = 4). (D, E) Effects of ISO preconditioning on MDA (D) and GSH (E) levels of neurons after O/R (*n* = 4). (F, G) Effects of ISO pretreatment on MDA (F) and GSH (G) levels in the rat cortex after I/R (*n* = 4). The data are expressed as mean ± SD, **p < 0.01, and ***p < 0.001 by one‐way analysis of variance (ANOVA). DCFH‐DA, dichlorodihydrofluorescein diacetate; GSH, glutathione; I/R, ischemia–reperfusion; MDA, malondialdehyde; O/R, oxygen–glucose deprivation/re‐oxygenation.

To better understand how ISO preconditioning prevents cerebral I/R injury, we performed untargeted metabolomic experiments to investigate the metabolomic profiles of the cerebral cortex after interventions. The partial least squares discriminant analysis score plot showed good separation among the sham, MCAO, and ISO groups, suggesting I/R injury, and ISO treatment led to alterations in corticocerebral metabolites (Figure 2A). The MCAO and sham groups had 214 shared metabolites, whereas the ISO and MCAO groups had 205 shared metabolites (Figure 2B,C). One hundred nineteen shared metabolites were identified among the three groups (Figure 2D,E). The functions of these differential metabolites were enriched and acquired by Kyoto Encyclopedia of Genes and Genomes analysis, and the results revealed that oxidative phosphorylation (OXPHOS) was significantly enriched (Figure 2F,G), hinting that the regulation of oxidative stress may be involved in ISO‐mediated neuroprotection on cerebral I/R injury. To explore this, we evaluated the effects of ISO preconditioning on OXPHOS in in vitro and in vivo studies (Figure 3A–C). The antioxidant effects were determined by measuring the MMP, and the oxidative stress was reflected by the level of ROS using the DCFH‐DA probe 6 h after O/R. O/R decreased the MMP of primary neurons; however, ISO pretreatment reversed the decrease. ROS, GSH, and MDA levels increased, compared with those of the control group, which were reversed by ISO preconditioning (Figure 3D,E). Consistent with the in vitro data, GSH and MDA levels in the cerebral cortex increased 6 h after I/R injury. In contrast, ISO pretreatment reduced GSH and MDA levels in the cerebral cortex (Figure 3F,G).

**FIGURE 4:**
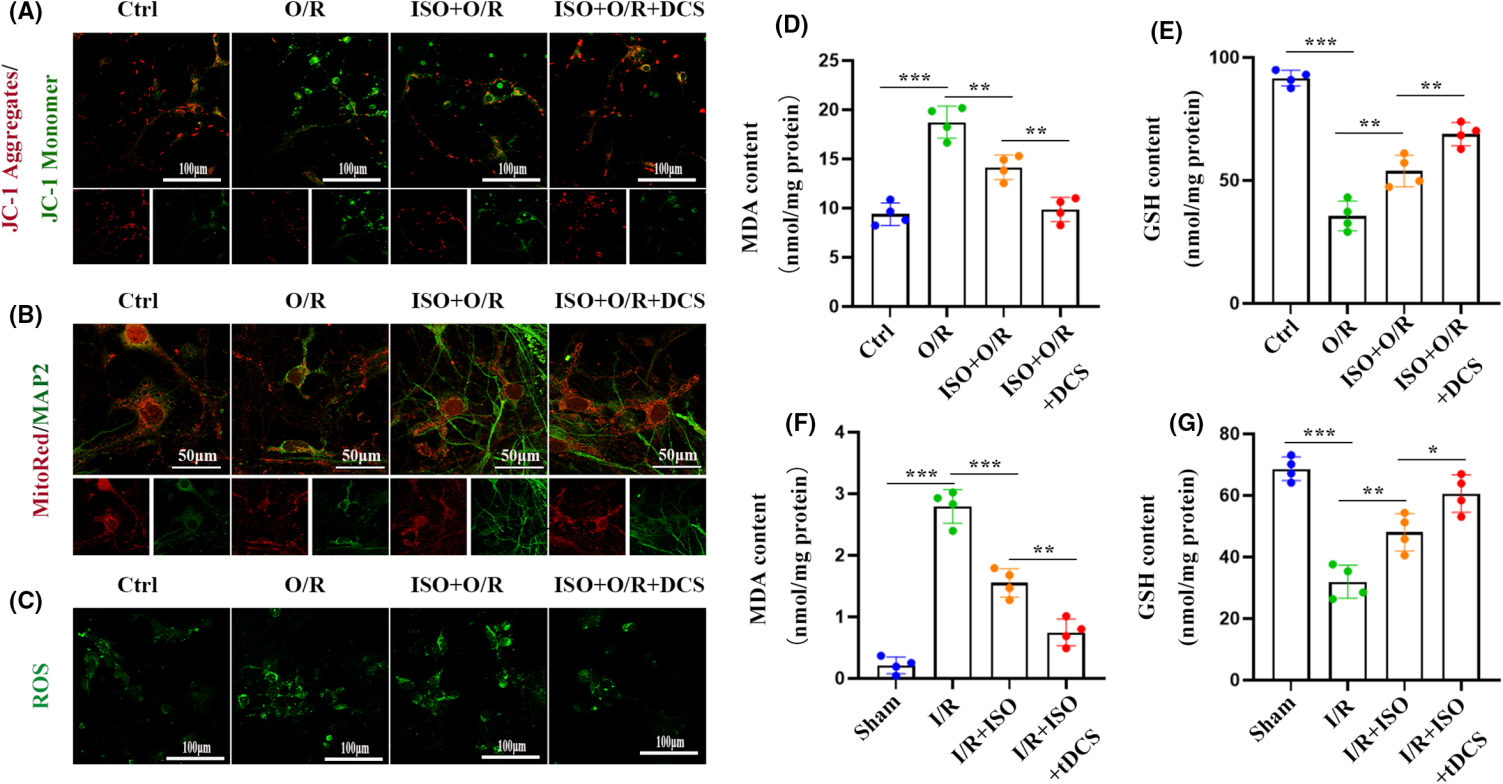
Transcranial direct current stimulation (tDCS) treatment can enhance ISO to further inhibit oxidative stress and neuroinflammation. (A) Using confocal neurons in JC‐1 aggregates (red) and monomers (green) (*n* = 4). (B) MitoTracker Red staining displays a representative image of neurons following ISO preconditioning, with neurons labeled as MAP2 (green) (*n* = 4). (C) The reactive oxygen species (ROS) levels of neurons labeled with DCFH‐DA fluorescent probes were analyzed by fluorescence microscopy (*n* = 4). (D, E) Effects of ISO and DCS preconditioning on MDA (D) and GSH (E) levels of neurons after oxygen–glucose deprivation/re‐oxygenation (*n* = 4). (F, G) Effects of ISO and tDCS pretreatment on MDA (F) and GSH (G) levels in rat cortex after I/R (*n* = 4). The data are expressed as mean ± SD, *p < 0.05, **p < 0.01, and ***p < 0.001 by one‐way analysis of variance (ANOVA). DCFH‐DA, dichlorodihydrofluorescein diacetate; GSH, glutathione; I/R, ischemia–reperfusion; ISO, isoflurane; MDA, malondialdehyde.

To demonstrate the synergistic effect of tDCS on ISO‐mediated inhibition of OXPHOS, tDCS or DCS in combination with ISO preconditioning was used to perform in vivo or in vitro experiments and the above experiments were repeated. The results showed that the combination therapy markedly increased the MMP (Figure 4A–C), increased GSH content and decreased MDA (Figure 4D–G) levels in the primary neurons and cerebral cortex. Altogether, these results indicate that tDCS enhances the inhibitory effects of ISO preconditioning on OXPHOS during cerebral I/R injury, which may initially inhibit ischemic stroke progression.

### 3.3 ISO preconditioning reversed the decrease in nuclear protein Akirin2 following cerebral I/R injury

**FIGURE 5:**
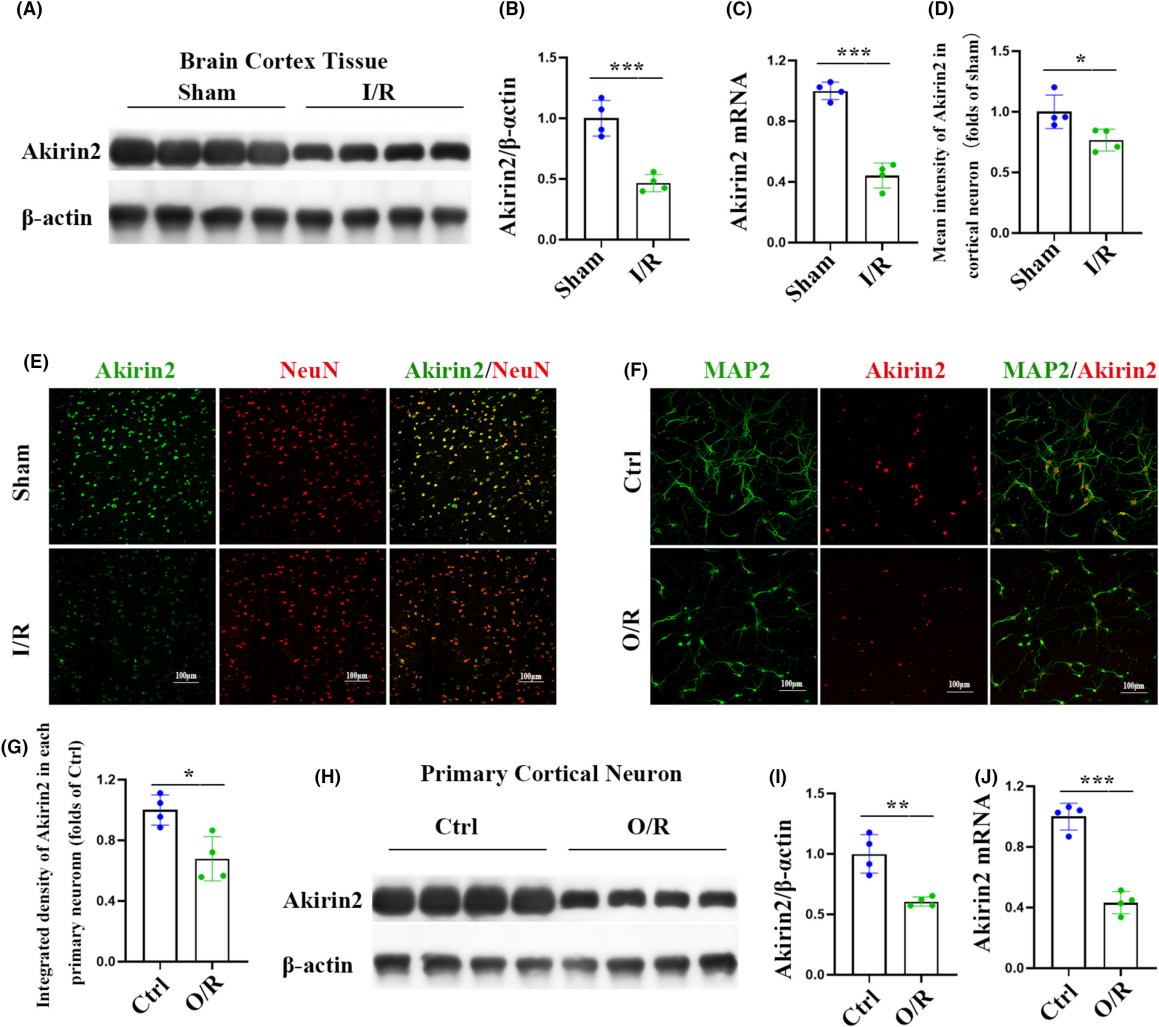
Akirin2 levels decrease in neurons following ischemia‐repression injury. (A) Representative Akirin2 western blotting images of rats 6 h after ischemia‐repression (I/R). (B, C) Western blot and qRT‐PCR analysis of Akirin2 in the cerebral cortex (*n* = 4). (D, E) Akirin2 immunofluorescence staining and quantification in the cerebral cortex. Neurons (NeuN, red) and Akirin2 (green) were stained 6 h after I/R (*n* = 4). (F, G) Akirin2 immunofluorescence staining and Quantification in neurons. Staining was performed 6 h after oxygen–glucose deprivation/re‐oxygenation. Akirin2 (red) is primarily detected within the nucleus, and neurons are labeled with MAP2 (green) (*n* = 4). (H) Representative Akirin2 western blotting images of neurons 6 h after O/R. (I, J) Western blot and qRT‐PCR analysis of Akirin2 in neurons (*n* = 4). The data are expressed as mean ± SD, *p < 0.05, **p < 0.01, and ***p < 0.001 by one‐way analysis of variance (ANOVA). qRT‐PCR, quantitative real‐time PCR.

**FIGURE 6:**
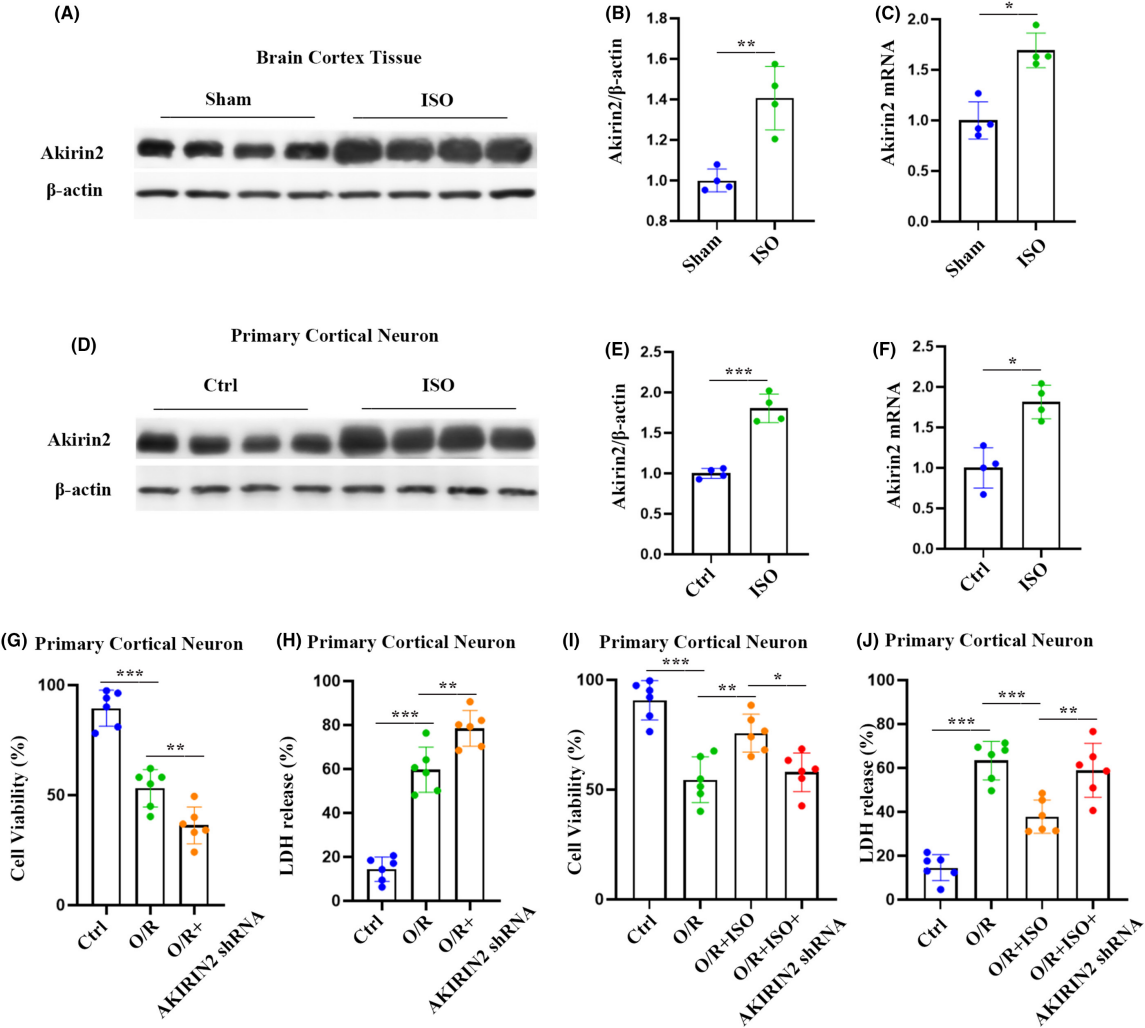
Isoflurane (ISO) upregulates Akirin2 and reduces oxidative stress and neuronal death following oxygen–glucose deprivation injury. (A) Representative Akirin2 western blotting images of ISO‐pretreated rat. (B, C) Western blot and qRT‐PCR analysis of Akirin2 in rats (*n* = 4). (D) Representative Akirin2 western blotting images of ISO‐pretreated neurons. (E, F) Western blot and qRT‐PCR analysis of Akirin2 in neurons (*n* = 4). Cell viability (G) and lactase dehydrogenase (LDH) release (H) were used to assess the effects of oxygen–glucose deprivation/re‐oxygenation (O/R) on neuronal survival. Cell viability or LDH release tests were performed 48 h after transfection of primary cultured neurons with shAkirin2 lentivirus (*n* = 6). Cell viability (I) and LDH release (J) were used to assess the effects of O/R on neuronal survival. Primary cultured neurons were transfected with shAkirin2 lentivirus for 48 h and pretreated with ISO for 6 h for cell viability or LDH release tests (*n* = 6). The data are expressed as mean ± SD, *p < 0.05, **p < 0.01, and ***p < 0.001 by one‐way analysis of variance (ANOVA). qRT‐PCR, quantitative real‐time PCR.

The nuclear protein Akirin2 is broadly expressed in the CNS and closely associated with the control of cell proliferation and differentiation, which is emerging as a critical regulator in neurological disorders.^11^, ^18^ As an initial step to investigate the possible molecular and cellular mechanism of ISO preconditioning for I/R injury, we examined the mRNA and protein levels of Akirin2 in the cerebral cortex and primary cortical neurons. As shown in Figure 5A–C, both the protein and mRNA expression of Akirin2 in the cerebral cortex decreased 6 h after I/R injury. The immunostaining results also showed a significant decrease in the expression of Akirin2^+^ in NeuN^+^ cells in the cerebral cortex in rats with I/R injury, compared with that in the sham group (Figure 5D,E). Consistent with the in vivo data, Akirin2 expression in primary cortical neurons showed similar reduction following OGD injury, evidenced by the decrease in Akirin2^+^ and MAP2^+^ co‐labeling cells, protein, and mRNA expression in primary cortical neurons (Figure 5F–J). We further investigated whether ISO affects neuronal Akirin2 expression using in vivo and in vitro models under physiological conditions. We measured the mRNA and protein levels of Akirin2 in the cerebral cortex and primary cortical neurons following ISO treatment. A 6‐h ISO treatment of normal rats significantly increased protein and mRNA expression in the cerebral cortex and primary cortical neurons (Figure 6A–F). Moreover, downregulation of Akirin2 deteriorated cell viability and promoted LDH release in primary cortical neurons subjected to O/R injury (Figure 6G,H). We further discovered that knockdown of Akirin2 reversed the ISO‐mediated therapeutic effect on survival after O/R injury (Figure 6I,J). Taken together, Akirin2 may maintain neuronal survival to promote recovery after cerebral I/R injury.

### 3.4 ISO exerts neuroprotection following cerebral I/R injury by targeting Akirin2

**FIGURE 7:**
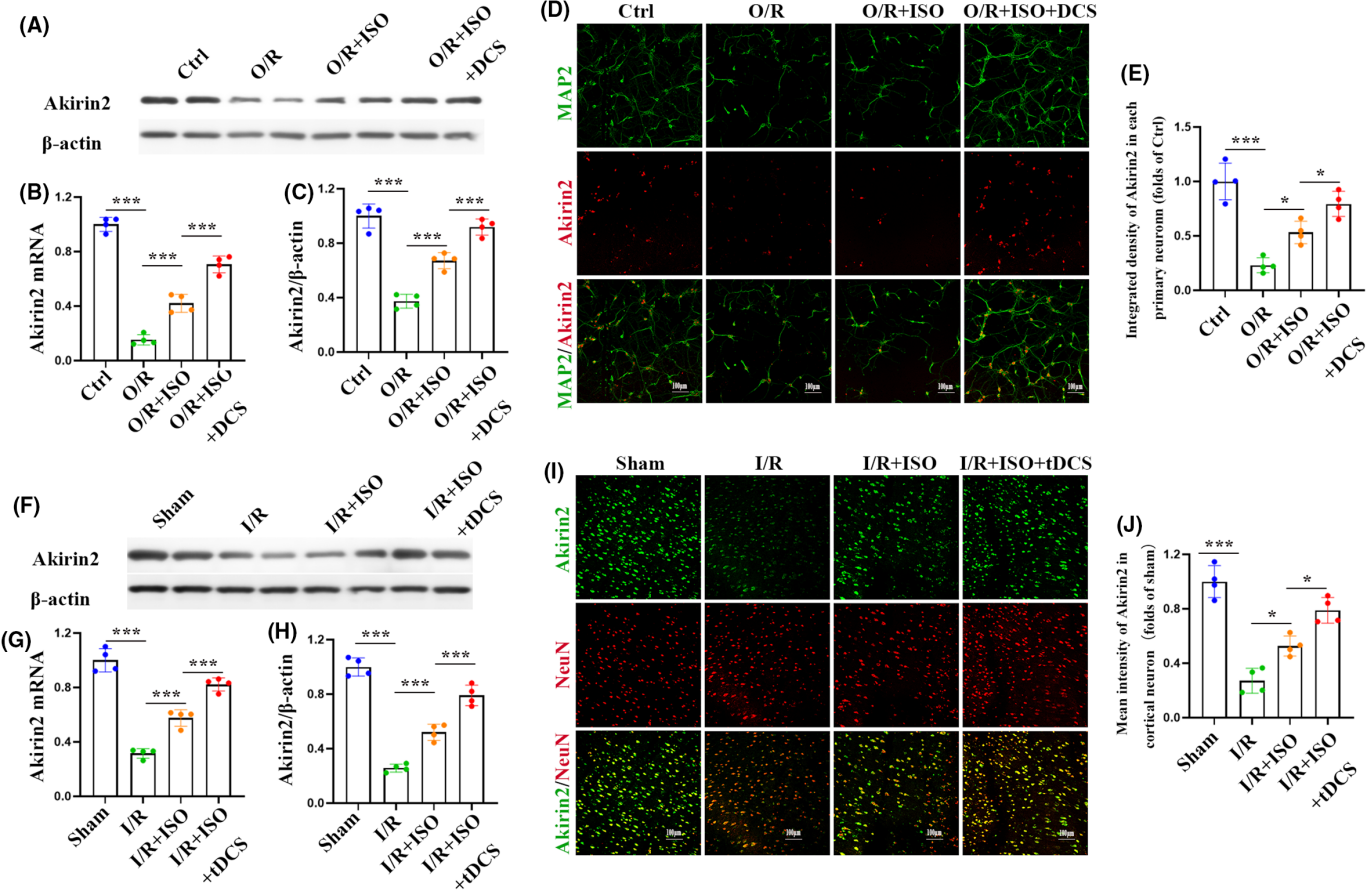
Transcranial direct current stimulation (tDCS) enhances the neuroprotective effect of isoflurane (ISO)‐mediated cerebral ischemia–reperfusion injury in rats by increasing the expression of Akirin2. (A) Representative western blotting images of neuronal Akirin2 after ISO and direct current stimulation pretreatment. (B, C) qRT‐PCR and western blotting analysis of Akirin2 protein expression in neurons (*n* = 4). (D) Representative images and Quantification of neuronal Akirin2 immunofluorescence in the primary neurons (*n* = 4). (F) Representative western blotting images of Akirin2 in cerebral cortex after ISO and tDCS pretreatment. (G, H) qRT‐PCR and western blotting analysis of Akirin2 protein expression in cerebral cortex after ISO and tDCS pretreatment (*n* = 4). (I) Representative images and (J) quantification of Akirin2 immunofluorescence in the cerebral cortex (*n* = 4). The data are expressed as mean ± SD, *p < 0.05 and ***p < 0.001 by one‐way analysis of variance (ANOVA). qRT‐PCR, quantitative real‐time PCR.

Subsequently, we explored how intraneuronal ISO mediates the reduction of neuronal death post‐ischemia–reperfusion injury through Akirin2. Primary cortical neurons were pretreated with ISO and DCS for 6 h before O/R. Following this, the cells were harvested to assess the mRNA and protein expression levels of Akirin2 after the 6‐h period. Our results showed that the combination therapy significantly suppressed both mRNA and protein expression of Akirin2, compared with that of ISO preconditioning alone (Figure 7A–C). Immunostaining for Akirin2 and MAP2 in primary neurons further confirmed an increase in neuronal Akirin2 in the ISO and DCS combination therapy group, compared with that in the ISO group (Figure 7D,E). MCAO was performed 6 h after tDCS and ISO preconditioning combination treatment in rats. The cerebral cortex was harvested to evaluate Akirin2 expression. Similar to the finding in in vitro experiments, ISO preconditioning and tDCS combination therapy promoted Akirin2 expression in neurons of the cerebral cortex, compared with that of ISO preconditioning. Moreover, mRNA and protein expression of Akirin2 were higher in the combination therapy that those in the ISO preconditioning group (Figure 7F–J). These results suggest that tDCS in combination with ISO preconditioning may attenuate neuronal damage and promote recovery after I/R injury via activating an Akinrin2‐related signaling pathway. This indicates that the combination therapy may be a potential intervention for ischemic stroke.

### 3.5 Akirin2‐functioned upstream of PTEN in cerebral I/R injury

**FIGURE 8:**
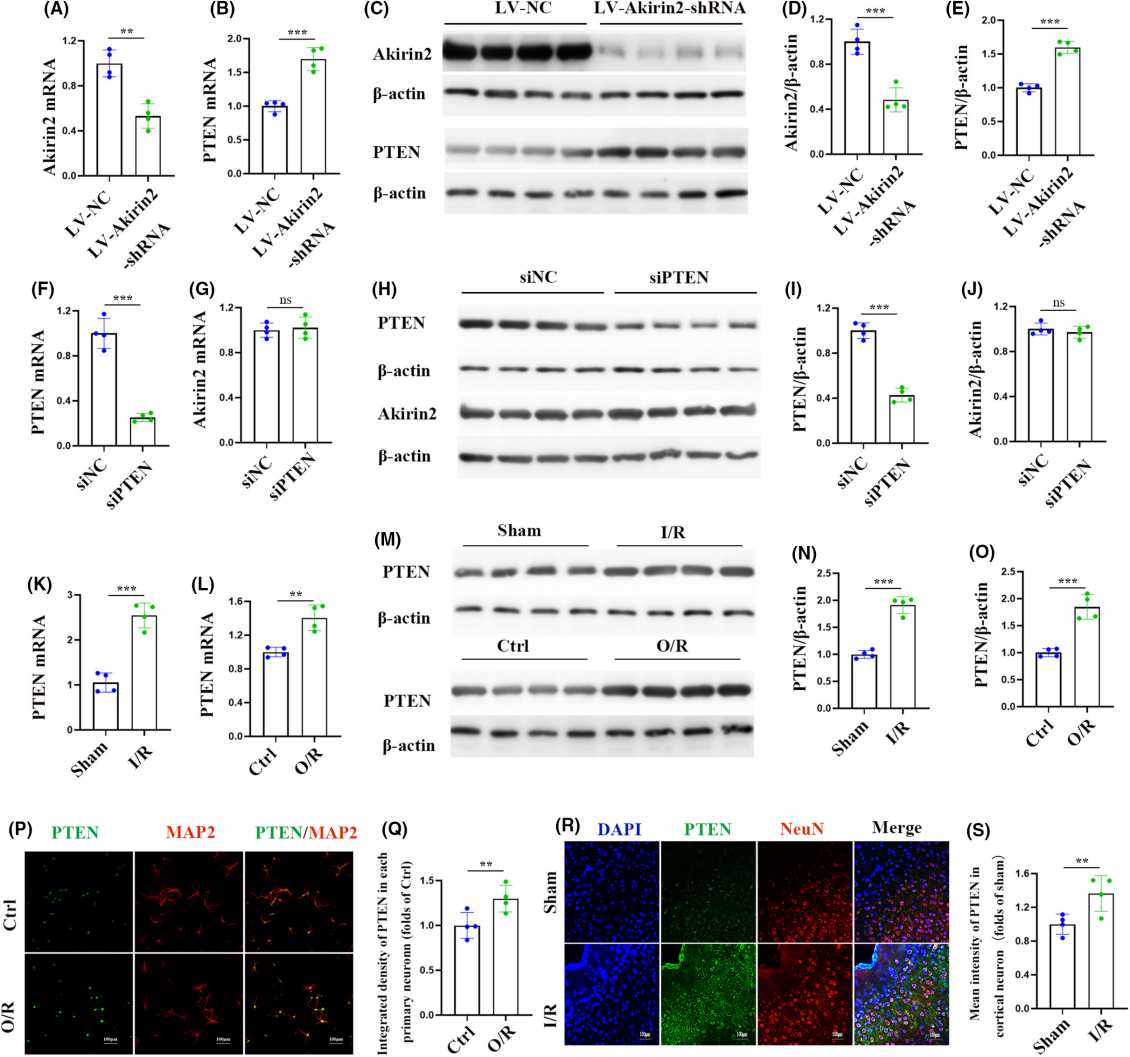
Downregulation of Akirin2 enhances PTEN expression following oxygen–glucose deprivation injury. (A, B) qRT‐PCR analysis of Akirin2 and PTEN mRNA expression in neurons 48 h after injection of LV‐NC or LV‐Akirin2 lentivirus. (C) Representative western blotting images of Akirin2 and PTEN in neurons 48 h after injection of LV‐NC or LV‐Akirin2 lentivirus. (D, E) Western blotting analysis of Akirin2 and PTEN protein expression (*n* = 4). (F, G) qRT‐PCR analysis of PTEN and Akirin2 mRNA expression in neurons transfected with siNC or siPTEN after 48 h. (H) Representative western blotting images of PTEN and Akirin2 in neurons transfected with siNC or siPTEN after 48 h. (I, J) Western blotting analysis of PTEN and Akirin2 protein expression (*n* = 4). (K, L) qRT‐PCR analysis of PTEN mRNA expression 6 h after neuronal O/R and ischemia–reperfusion in the cerebral cortex and neurons (*n* = 4). (M) Representative western blotting images of PTEN protein expression 6 h after neuronal O/R and rat ischemia–reperfusion in neurons and the cerebral cortex. (N, O) western blot analysis of PTEN in the cerebral cortex and neurons (*n* = 4). (P) Representative images of PTEN immunofluorescence in neurons after oxygen–glucose deprivation injury. PTEN (green), MAP2‐labeled neurons (red). (Q) Quantifcation of integrated density of PTEN in the primary neurons (*n* = 4). (R) Representative images of PTEN immunofluorescence in the cerebral cortex after I/R injury. PTEN (green), NeuN‐labeled neurons (red). (S) Quantification of PTEN intensity in cortical neurons (*n* = 4). The data are expressed as mean ± SD, **p < 0.01, and ***p < 0.001 by one‐way analysis of variance (ANOVA). ns, non‐significant; PTEN, phosphatase and tensin homolog deleted on chromosome 10; qRT‐PCR, quantitative real‐time PCR.

**FIGURE 9:**
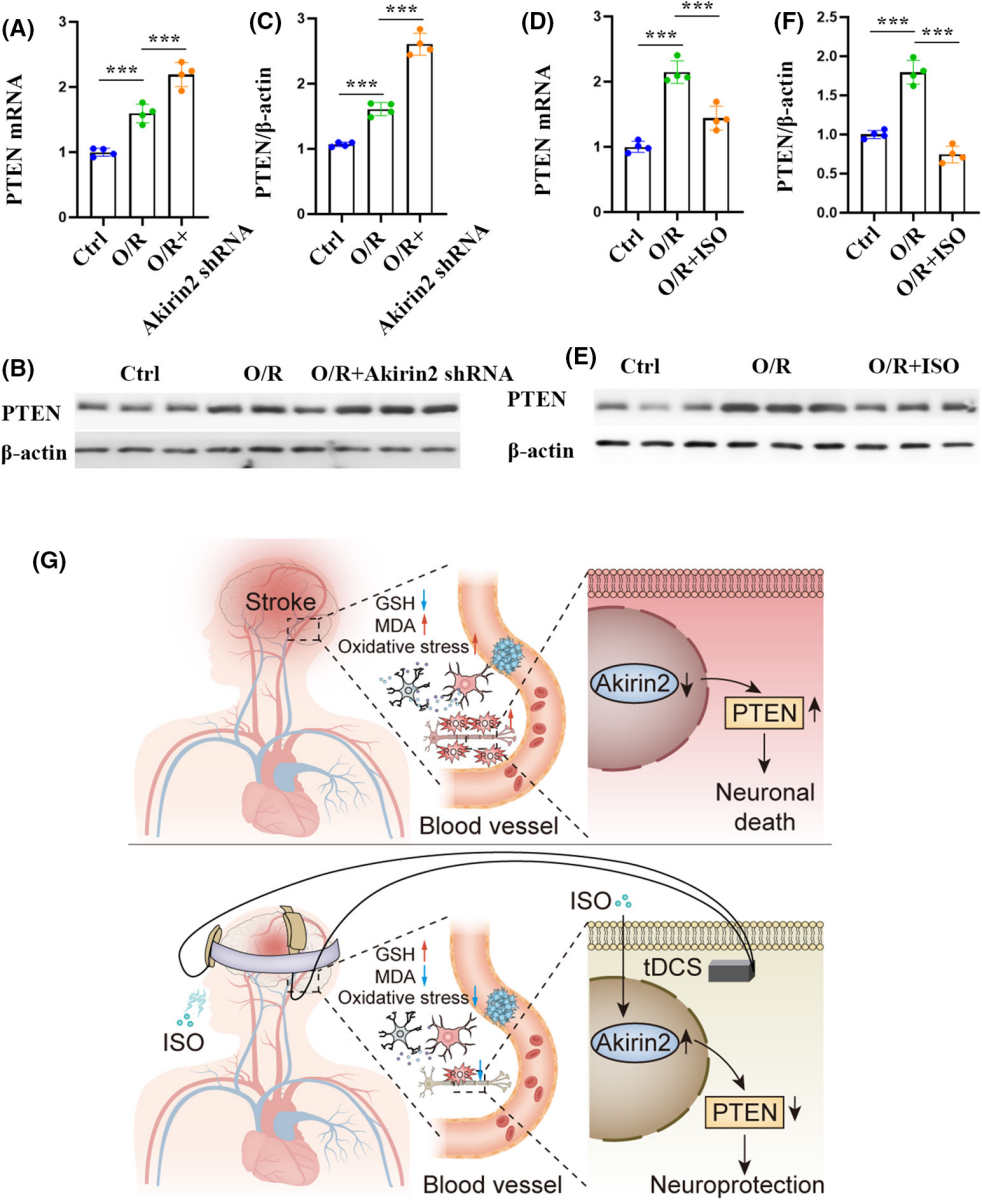
Isoflurane (ISO) promotes Akirin2 upregulation and PTEN downregulation within the neurons following oxygen–glucose deprivation injury. (A) qRT‐PCR analysis of PTEN mRNA expression in neurons transfected with shAkirin2 lentivirus for 48 h. (B) Representative western blotting images of PTEN in neurons transfected with shAkirin2 lentivirus for 48 h. (C) Western blotting quantitative analysis of PTEN protein expression in neurons (*n* = 4). (D) qRT‐PCR analysis of PTEN mRNA expression in neurons pretreated with ISO. (E) Representative western blotting images of PTEN in neurons pretreated with ISO. (F) Western blotting quantitative analysis of PTEN protein expression in neurons (*n* = 4). The data are expressed as mean ± SD, ***p < 0.001 by one‐way analysis of variance (ANOVA). (G) Schematic representation of transcranial direct current stimulation (tDCS) enhancing the effects of ISO preconditioning on ischemic stroke. Ischemic stroke triggers oxidative stress in the cerebral cortex and neurons. The progression of ischemic stroke decreases neuronal Akirin2 expression, leading to PTEN upregulation, neuronal death, and brain damage. tDCS boosts the protective impact of ISO preconditioning on oxidative stress and activates the Akirin2‐PTEN signaling pathway to facilitate neuronal recovery. These combined mechanisms play a pivotal role in brain injury recovery post‐ischemic stroke. PTEN, phosphatase and tensin homolog deleted on chromosome 10; qRT‐PCR, quantitative real‐time PCR.

We previously found that overexpression of phosphatase and PTEN was involved in the pathological process for ischemic stroke.^24^ Considering that PTEN elevation increased mitochondrial OXPHOS and inhibited neuronal survival,^38^, ^39^ we examined whether Akirin2 interacts with PTEN in the context of I/R injury. To explore this, we first knocked out Akirin2 in primary cortical neurons by infecting it with concentrated lentivirus. Akirin2 knockdown was confirmed by qPCR and western blot (Figure 8A,C,D). The results showed a significant PTEN decrease in Akinrin2 knockdown primary neurons (Figure 8B,C,E). However, the knockdown of PTEN by siRNA delivery did not affect Akirin2 expression (Figure 8F–J), which implies that Akirin2 may be the upstream of PTEN. Next, we investigated the role of the Akirin2‐PTEN signaling pathway in cerebral I/R injury and ISO preconditioning. Both the mRNA and protein expression of PTEN in the cerebral cortex and primary cortical neurons decreased after I/R or O/R injury. We also examined the PTEN levels in neurons by immunostaining 6 h after O/R and cerebral I/R injury (Figure 8K–O). As shown in Figure 8P–S, stronger PTEN signals were observed in the neurons and cortex in the O/R or I/R groups, and the expressions of PTEN^+^ in MAP2^+^ and NeuN^+^ cells significantly increased following the modeling process. Furthermore, we confirmed that Akirin2 was the upstream of PTEN in ischemic stroke, as evidenced by an increased expression of PTEN mRNA and protein after Akinrin2 knockdown in primary cortical neurons subjected to O/R injury (Figure 9A–C). Similarly, ISO preconditioning also inhibited the expression of PTEN mRNA and protein in primary cortical neurons subjected to O/R injury (Figure 9D–F). These results suggest that Akinrin2 can regulate PTEN expression in cerebral I/R injury and the Akirin2‐PTEN signaling pathway may be a mechanism for ISO preconditioning‐mediated neuroprotection against neuronal impairments in ischemic stroke.

## 4 DISCUSSION

Our results suggest that ISO pretreatment exerted a protective effect against I/R injury as manifested by the reduced infarct volume in rats with MCAO and reduced cortical neuronal death after O/R injury. We hereby present for the first time that cathodal tDCS enhanced the neuroprotective effect of ISO preconditioning against neuronal injury and improved neurological outcomes after I/R injury. Metabolomics profiling analysis revealed that OXPHOS was involved in the pathogenesis of I/R injury. Consistent with the above functional data, tDCS in combination with ISO preconditioning further attenuated oxidative stress to I/R injury. The combination therapy protected against I/R injury by inhibiting oxidative stress and upregulating Akirin2 expression, thus downregulating its potential downstream PTEN in the cortex and in the primary neurons. This suggests that the Akirin2‐PTEN signaling pathway may, at least in part, be a potential mechanism underlying the neuroprotective effect of tDCS and ISO in cerebral ischemia/reperfusion injury.

Volatile anesthetics, such as ISO and sevoflurane, have been reported to have neuroprotective effects against cerebral ischemia, and this is particularly valuable for preventing ischemic stroke in the perioperative setting.^40^ A preclinical study demonstrated neuroprotection by repeated sevoflurane preconditioning against brain damage after ischemic stroke for up to 3 days.^41^ Significant prophylactic neuroprotective effects of volatile anesthetics against ischemic stroke on patients undergoing noncardiac surgery with general anesthesia are observed until postoperative day 17.^6^ Simultaneously, the clinical study also demonstrated the therapeutic dose‐dependency of volatile anesthetics on stroke incidence and severity. Compared with lower concentrations of volatile anesthetics, higher concentrations were more likely to prevent ischemic stroke.^6^ However, the conclusion remains uncertain because of conflicting evidence. Other studies showed that low doses of ISO could exhibit quicker brain protection against ischemic stroke; however, little neuroprotection, and even worsening effects, at high doses was observed.^42^ Furthermore, short treatment durations of ISO limit its therapeutic effect.^8^ Therefore, further investigation is warranted on the effectiveness of ISO preconditioning on stroke, and combination therapy may provide a new therapeutic opportunity.

Transcranial direct current stimulation is a safe, non‐invasive, and low‐cost brain stimulation neuromodulation technique.^43^ It applies low‐intensity (1–2 mA) DC using bioconducting electrodes to exert a polarity‐specific neuromodulation, regulates cortical excitability and spontaneous neural activity, and enhances functional connectivity strength of key nodes in underlying brain regions.^43^, ^44^ Evidence suggests that tDCS is beneficial in multiple neurological and psychiatric disorders, including depression, Parkinson's disease, epilepsy, schizophrenia, and alcohol addiction, despite showing different levels of therapeutic efficacy.^45^ tDCS has recently been identified as a promising approach to reverse stroke‐induced network alterations, enhance neuroplasticity, and accelerate functional recovery after stroke in animal models.^10^, ^46^ Clinical evidence showed similar therapeutic effects of tDCS on neuroplasticity and network connectivity of the motor cortex following stroke.^47^ tDCS application in the acute stages of stroke was also explored and the functional recovery was found to be not only accelerated but also improved. The efficiency can be maintained up to 1 year after stroke,^48^ indicating that an earlier intervention may help decrease the incidence and severity of ischemic stroke. tDCS as an effective treatment is being explored. A randomized controlled trial has shown that one session of anodal tDCS over the left dorsolateral prefrontal cortex may be associated with a lower incidence of postoperative delirium after major surgery in elderly patients when used during the perioperative period.^44^ Patients with oral cancer could clinically benefit from its postoperative relief of depression and anxiety.^49^ However, tDCS protocols to effectively enhance the therapeutic effects of ISO preconditioning and improve ischemic stroke are still lacking. Furthermore, enough mechanistic neurobiological understanding will promote its clinical translation. Our data show that tDCS pretreatment can be treated similarly to ISO pretreatment compared with rats pretreated with ISO alone and that rats pretreated with a combination of tDCS and ISO have better recovery in terms of brain injury and neurological outcomes. Similarly, the survival of cortical neurons improved after DCS in in vitro neurons administered with ISO. These results provide direct evidence that tDCS may facilitate ISO preconditioning‐mediated effects on recovery of ischemic stroke.

A better understanding of the neurobiological mechanisms underlying how the combination therapy helps repair damaged neurons after oxygen and energy depletion would provide a foundation to the translation of the approach as a treatment for ischemic stroke. Maintaining neurogenesis status requires the continuous production of neurons from neural progenitor cells once neurons are damaged or lost after stroke.^50^ Akirin2, a highly conserved nuclear protein that interacts with transcription factors to regulate gene expression, is critical for the normal proliferation, differentiation, and survival of cortical progenitor cells by regulating cell cycle progression and inhibiting apoptosis.^17^ A recent study suggests an essential role of Akirin2 in maintaining healthy neurons during cortical maturation and that Akirin2 dysfunction may lead to neurodegenerative diseases.^18^ Our results suggest that rat primary neurons exhibited a significant decrease in Akirin2 expression after stroke, indicating that Akirin2 may be involved in the development of ischemic stroke. An important molecule downstream of Akirin2 to induce the effect on cerebral ischemia/reperfusion injury may be PTEN signaling. Oxidative stress is considered critical pathological processes for ischemic stroke.^51^ The untargeted metabolomic data, along with our in vitro and in vivo findings, underscored the crucial role of OXPHOS in ischemic stroke (Figure 2). PTEN has been recognized as a critical cancer inhibitor since its discovery,^52^ and as shown by Wan et al., GABA increased neuron survival by preserving the function of type A receptors after stroke when downregulated.^53^ Notably, cells derived from “Super‐PTEN” mutant mice exhibit increased mitochondrial OXPHOS.^38^ Akirin2 activation decreased PTEN expression, whereas PTEN activation failed to affect Akirin2 expression in our studies. More importantly, tDCS and ISO pretreatment reversed the I/R injury‐induced downregulation of Akirin2. These results suggest that PTEN is the critical downstream signaling of Akirin2 in I/R injury. tDCS enhances ISO‐mediated neuroprotective effects by regulating Akirin2‐PTEN signaling.

This study has significant implications for clinical translation. Our findings provided direct evidence that tDCS can effectively enhance the neuroprotective potential of ISO on ischemic stroke. This promotes the shift of ischemic stroke intervention from monotherapy to combination or multiple therapies, given that the synergistic therapeutic effect of different techniques may result in unexpected therapeutic effects in hard‐to‐treat diseases. This is especially valuable because volatile anesthetics are widely applied in clinics. Moreover, we report for the first time that the nuclear protein Akirin2 plays an important role in the pathogenesis of ischemic stroke and tDCS in combination with ISO preconditioning‐mediated neuroprotective effects. Therefore, Akirin2 may act as a new therapeutic target to prevent and treat cerebral ischemia/reperfusion injury. However, although we determined the possible neuroprotective effects of the Akirin2‐PTEN signaling pathway on recovery of ischemic stroke, the detailed mechanisms for the signaling to induce these effects were not determined. For example, we did not determine whether the Akirin2‐PTEN pathway can regulate the proliferation, migration, differentiation, and survival of neural progenitor cells.

## 5 CONCLUSIONS

Our study indicates that ISO preconditioning can reduce neurological damage following ischemic stroke, and a more effective therapeutic outcome can be achieved when combined with tDCS. The therapeutic benefits of combining ISO preconditioning with tDCS may, at least in part, stem from the inhibition of OXPHOS and activation of the Akirin2/PTEN signaling pathway. The study offers a novel intervention strategy for the prevention and treatment of ischemic stroke.

## AUTHOR CONTRIBUTIONS

XK and PW performed the experiment and wrote the manuscript. WL, XL, performed the in vivo experiments. LX, HF, CL, XS, CL performed analyzed the data. JL and PW conceived the project and revised the manuscript.

## FUNDING INFORMATION

Support for this study was provided from the Natural Science Foundation of Shandong Province (ZR2020QH291 and ZR2020MH126), the Qingdao Key Health Discipline Development Fund (QDZDZK2022094), the Scientific Research Foundation of Qilu Hospital of Shandong University (QDKY2023ZD02) and the Qingdao Outstanding Health Professional Development Fund (2023).

## CONFLICT OF INTEREST STATEMENT

The authors declare that they have no competing interests.

## Supporting information


Data S1.

## Data Availability

The metabolomic data used and analyzed during the current study are publicly available in the MetaboLights repository at https://www.ebi.ac.uk/metabolights/editor/study/MTBLS8818. Other datasets used and analyzed during this study can be obtained from the corresponding author upon reasonable request.
