# Supplementary figures and images for: Transcranial direct current stimulation enhances the protective effect of isoflurane preconditioning on cerebral ischemia/reperfusion injury: A new mechanism associated with the nuclear protein Akirin2
Source: CNS Neurosci Ther. 2024 Sep 12;30(9):e70033. doi: 10.1111/cns.70033 (PMC11393012; doi:10.1111/cns.70033)

Full unedited gelblot for Figure 5a

$\beta$ -actin  
42 kDa←

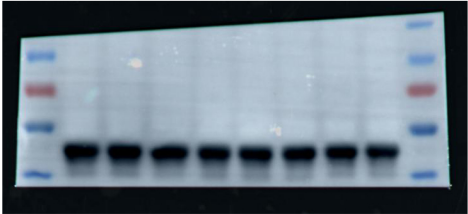

→55 kDa  
→40 kDa

35 kDa←  
Akirin2

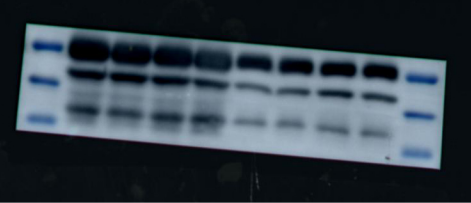

→35 kDa  
→25 kDa

Supplement: Supplementary file 1 — Data S1. [file CNS-30-e70033-s001.zip › Fig 5a.pdf]

Full unedited gelblot for Figure 5h

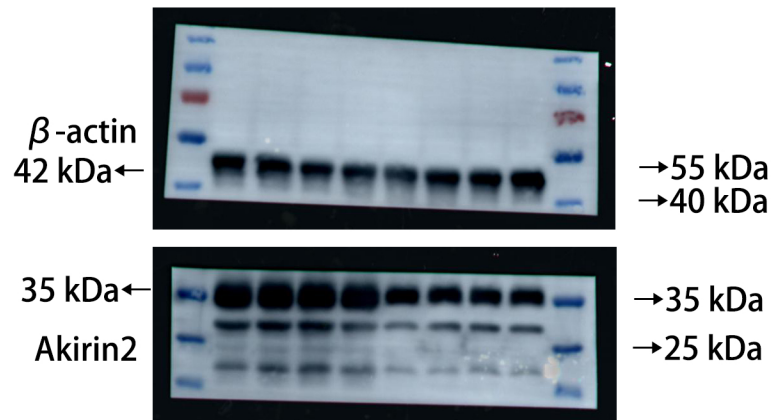

Supplement: Supplementary file 1 — Data S1. [file CNS-30-e70033-s001.zip › Fig 5h.pdf]

Full unedited gelblot for Figure 6a

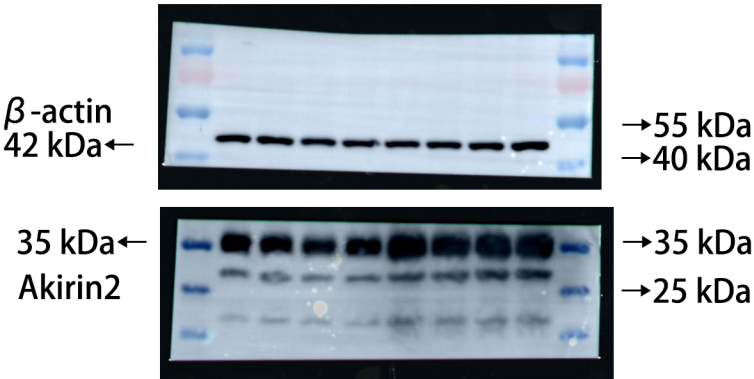

Supplement: Supplementary file 1 — Data S1. [file CNS-30-e70033-s001.zip › Fig 6a.pdf]

Full unedited gelblot for Figure 6d

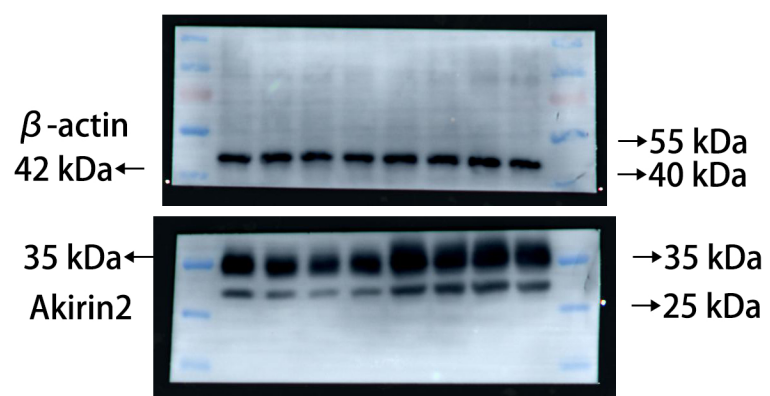

Supplement: Supplementary file 1 — Data S1. [file CNS-30-e70033-s001.zip › Fig 6d.pdf]

Full unedited gelblot for Figure 7a

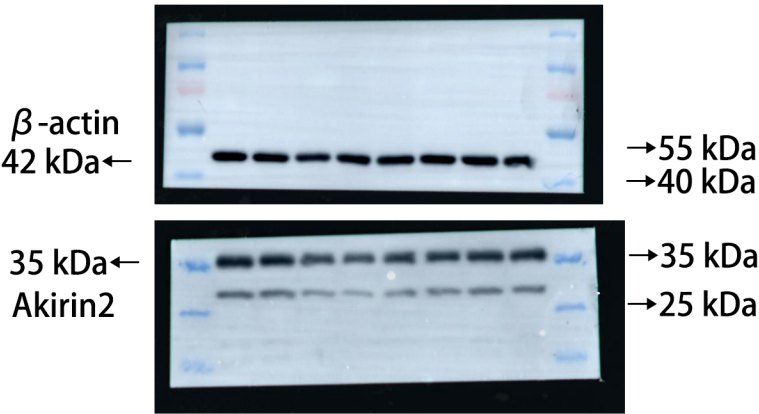

Supplement: Supplementary file 1 — Data S1. [file CNS-30-e70033-s001.zip › Fig 7a.pdf]

Full unedited gelblot for Figure 7f

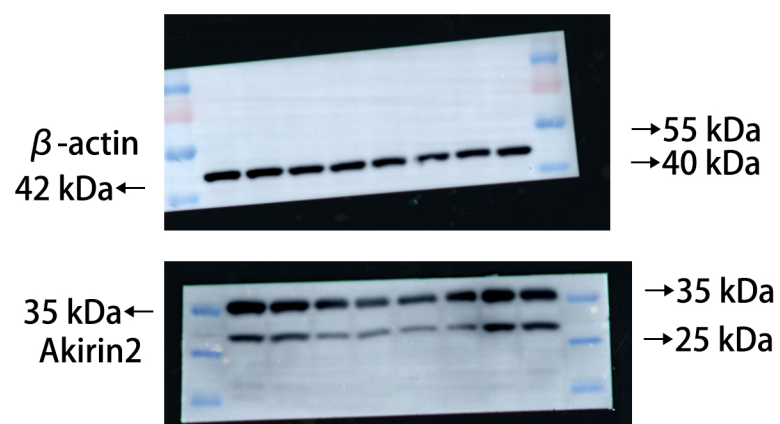

Supplement: Supplementary file 1 — Data S1. [file CNS-30-e70033-s001.zip › Fig 7f.pdf]

Full unedited gelblot for Figure 8c

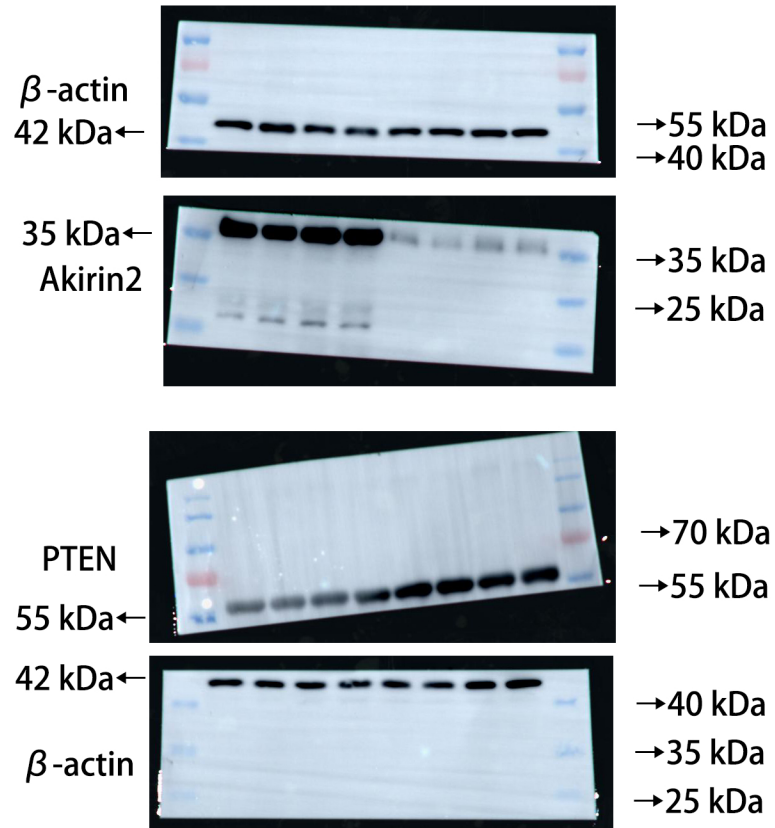

Supplement: Supplementary file 1 — Data S1. [file CNS-30-e70033-s001.zip › Fig 8c.pdf]

Full unedited gelblot for Figure 8h

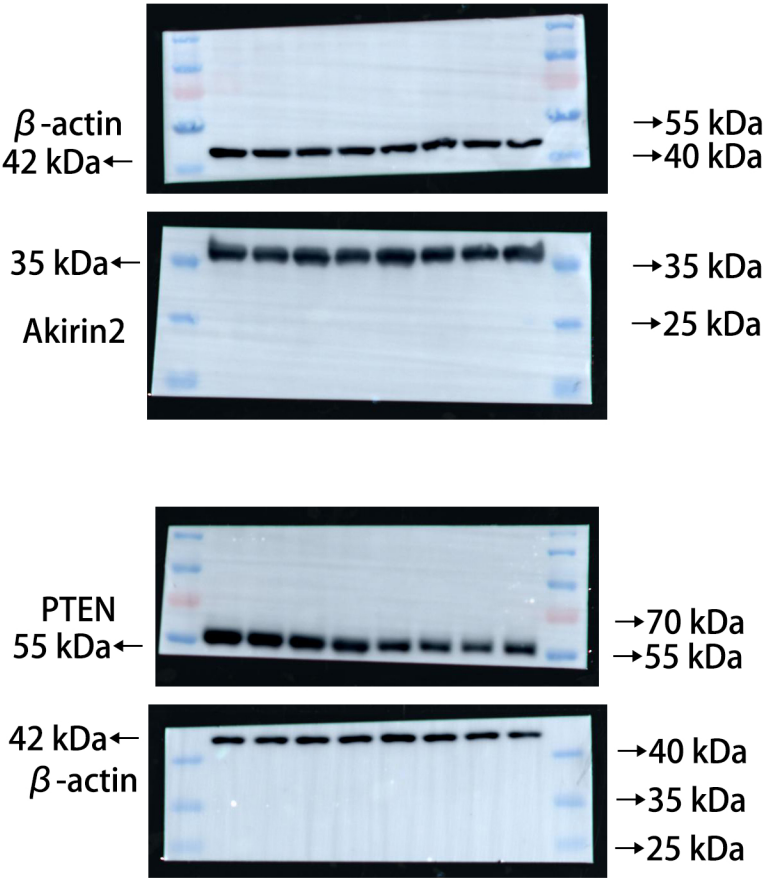

Supplement: Supplementary file 1 — Data S1. [file CNS-30-e70033-s001.zip › Fig 8h.pdf]

Full unedited gelblot for Figure 8m

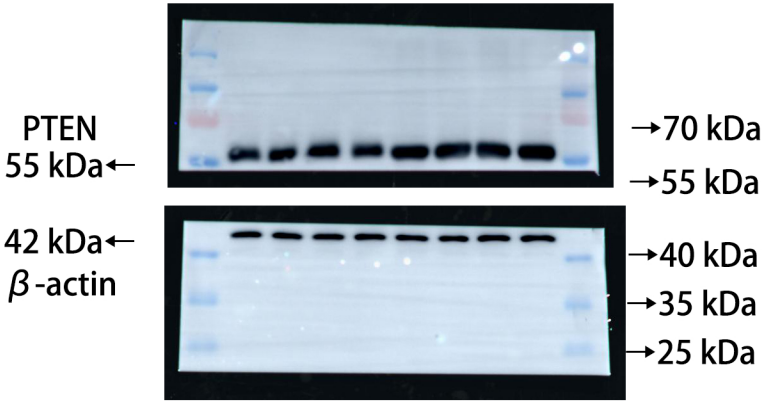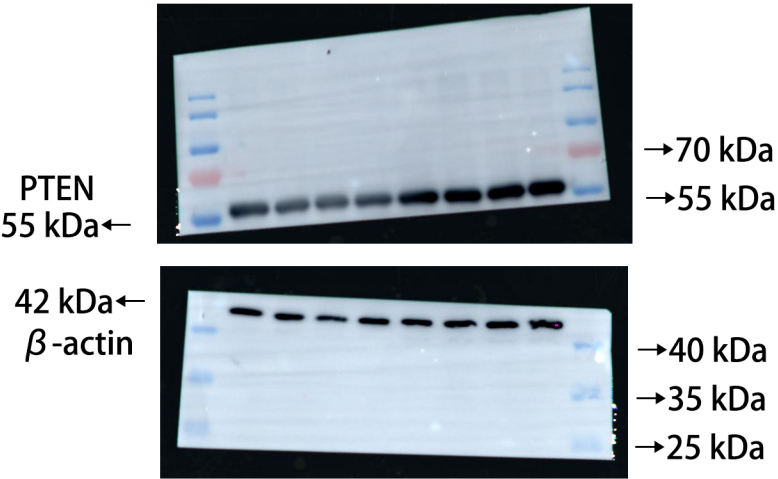

Supplement: Supplementary file 1 — Data S1. [file CNS-30-e70033-s001.zip › Fig 8m.pdf]

Full unedited gelblot for Figure 9b

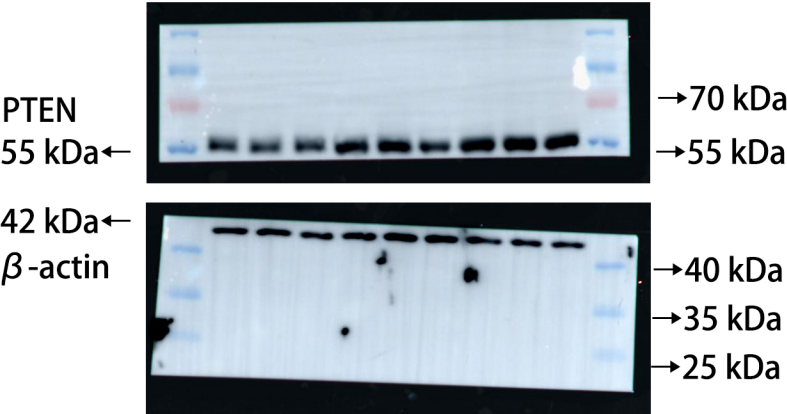

Supplement: Supplementary file 1 — Data S1. [file CNS-30-e70033-s001.zip › Fig 9b.pdf]

FigFull unedited gelblot for Figure 9e

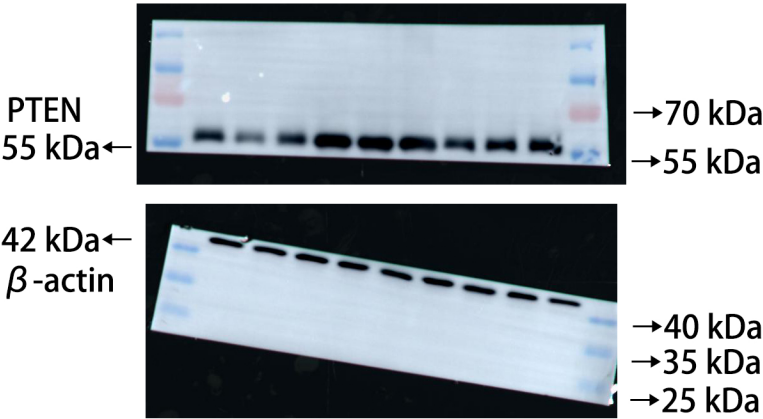

Supplement: Supplementary file 1 — Data S1. [file CNS-30-e70033-s001.zip › Fig 9e.pdf]
